# The Effect of Citrate Plasticisers TBC and ATBC on Biobased and Sustainable PHB-Based Polymer Blends

**DOI:** 10.3390/polym18131641

**Published:** 2026-07-01

**Authors:** Lorenzo Novembre, Luca Sconosciuto, Vito Emanuele Carofiglio, Domenico Centrone, Alessandro Sannino, Antonio Greco

**Affiliations:** 1 Department of Engineering for Innovation, University of Salento, 73100 Lecce, Italy; 2 EggPlant S.r.l., 00198 Rome, Italy; 3 IRCCS Istituto Tumori “Giovanni Paolo II”, 70124 Bari, Italy

**Keywords:** poly(3-hydroxybutyrate), polylactic acid, poly(butylene adipate-co-terephthalate), biopolymers, citrates, plasticisers, crystallisations, affinity

## Abstract

The development of fully biodegradable poly(3-hydroxybutyrate) (PHB)-based materials with improved mechanical performance remains a major challenge due to the limited ductility and processability of this highly crystalline polymer. Blending and plasticisation are viable strategies to enhance PHB toughness; however, the interactions governing polymer–plasticiser compatibility and their impact on structure–property relationships remain not fully understood. In this work, the compatibility and plasticisation mechanisms of two citrate-based plasticisers, tributyl citrate (TBC) and acetyl tributyl citrate (ATBC), were systematically investigated in biodegradable blends based on PHB, polylactic acid (PLA), and poly(butylene adipate-co-terephthalate) (PBAT). Polymer–plasticiser affinity was evaluated through Hansen Solubility Parameters and interaction radius, which indicated good compatibility of PHB with both plasticisers and a stronger affinity for ATBC. Differential scanning calorimetry showed that citrate plasticisers reduced the glass transition temperature, modified crystallisation kinetics, and altered the crystalline morphology of the blends. Dynamic mechanical analysis confirmed the reduction in the glass transition temperature of PHB–PLA systems, which is in agreement with the DSC results. Migration experiments showed equilibrium after approximately 72 h, with PHB–PLA blends exhibiting better plasticiser retention than PHB–PBAT systems. TBC consistently showed higher migration than ATBC, in line with its lower molecular weight and higher volatility. Mechanical testing demonstrated that plasticisation efficiency strongly depended on blend composition: TBC was more effective in enhancing ductility in PHB–PLA blends, whereas ATBC performed better in PHB–PBAT systems. It was also highlighted that the plasticisers had a remarkable ability to substantially increase the ductility of the blends compared with their unplasticised counterparts, as reflected by the pronounced decrease in stiffness and the marked increase in elongation at break. SEM analysis of tensile fracture surfaces evidenced a brittle failure mode for PHB–PLA blends, whereas PHB–PBAT systems exhibited a ductile fracture mode with fibrillar features and clear signs of phase separation. Finally, thermogravimetric analysis showed no appreciable thermal degradation within the processing temperature window used for mixing and hot pressing, confirming the thermal stability of the materials under the selected conditions. These findings establish clear correlations between thermodynamic compatibility, migration behaviour, thermal properties, fracture mechanisms, and mechanical performance, providing useful guidelines for the design of citrate-plasticised PHB-based biodegradable materials.

## 1. Introduction

The rising awareness of an unsustainable production scheme, based on non-renewable resources exploitation, induces a rethinking of entire sectors such as energy, fuels, and materials. Academics, institutions, companies and everyday customers find the bioplastics theme of particular interest, which involves the utilisation of new resources, innovative production approaches, new properties and applications, up to a different end-of-life of the product. Plastics, which are used daily for numerous applications, not only come from unsustainable raw materials that are facing progressive depletion and actively contribute to intensifying greenhouse gas emissions but are also becoming increasingly difficult and complex to manage once their use has ended [1]. In a single-use approach, plastics fulfil their designated functions impeccably, yet they are discarded immediately once their use has been completed. This vicious circle of “mass production–single-use–disposal” has become an automatic process in modern society, which, although undeniably providing benefits and comfort in the short term, has disastrous side effects from an environmental perspective due to the long degradation times of conventional plastics, from 100 to 1000 years [2].

To overcome the problems generated by fossil plastic, academia and industry are focusing their interest on biopolymers and other compostable and biodegradable materials.

Among several materials categorised as bioplastics, the family known as polyhydroxyalkanoates (PHA) seems to be an excellent and valid choice for a sustainable future, due to potentially wide applications based on the numerous properties of different PHA types [3].

Within this class of polymers, poly(3-hydroxybutyrate) (PHB), a biodegradable thermoplastic polyester, is considered a potential and valid substitute for synthetic polymers in many applications due to its excellent qualities. PHB is naturally produced as an energy storage material within microorganisms and bacteria, from which it is then extracted, processed, and produced, typically in the form of pellets or granules [4].

The mentioned properties and the possibility to process PHB through extrusion, injection, blowing and thermoforming [5] create great interest in the polymer, which is often compared to polypropylene (PP) for its technical properties. At the moment, PHB is available on the market, but only in a small percentage, as every new product will require development in processes and cost optimisations. Industrial application is hindered by processing challenges, mainly identified in low thermal stability [5], high crystallinity [6] and ageing [7,8]. Thermal stability is influenced by a narrow processing window due to the proximity of PHB melting temperature (T_m_ ≈ 170–180 °C) to its degradation onset (T_deg_ ≈ 245–260 °C) [9]. These limitations result in poor melt strength and susceptibility to thermal degradation during standard processing techniques such as extrusion and injection moulding [10]. Crystallinity and ageing phenomena are linked: over time, secondary crystallisation occurs, and this results in a lamellar structural reorganisation, causing embrittlement that severely reduces its mechanical performance [11,12].

To overcome these disadvantages, various strategies can be employed, such as the addition of plasticisers, thermal treatments, blending with other polymers [13], inclusion of natural fibres or solid fillers, realising strengthened composite materials, or even modification of the polymer structures with chemical functionalisation [8].

Copolymerisation, particularly with 3-hydroxyvalerate (HV), is another effective strategy. Poly(3-hydroxybutyrate-co-3-hydroxyvalerate) (PHBV) copolymers exhibit reduced crystallinity and enhanced flexibility. Alfano [14] showed that increasing HV content from 3 to 28 mol% lowered the melting point from 175 °C to 100 °C and increased elongation at break from 7% to 120%. Barbosa [15] demonstrated that plasticisation of PHBV with oligomeric polyesters improved miscibility and reduced stiffness. Jin [16] reviewed PHBV copolymers and highlighted their improved degradation behaviour and mechanical flexibility. Morais [17] showed that blending PHBV with poly(3-hydroxybutyrate-co-4-hydroxybutyrate) improved thermal stability and impact resistance.

Plasticisation is another widely studied approach. The addition of plasticisers such as epoxidised linseed oil (ELO), maleinised linseed oil (MLO), and epoxidised fatty acid esters (EFAE) has been shown to improve ductility and reduce glass transition temperature, enhancing processability. Garcia-Garcia [9] demonstrated that 5 phr of EFAE increased elongation at break by 40% and impact strength by 109%, although higher concentrations led to phase separation. Rapa [18] found that plasticisers like dioctyl adipate (DOA) and triacetyl glycerol (TAG) improved melt processability and reduced brittleness. Grillo Fernandes [19] showed that PEG and glycerol reduce the glass transition temperature but may compromise thermal stability. Iglesias Montes [20] investigated poly[di(ethylene glycol) adipate] in PLA/PHB blends, showing improved flexibility and compostability. Sousa Junior [21] demonstrated that linear-chain polyester oligomers reduced PHB’s elastic modulus by 72% and increased elongation at break by 467%.

To overcome PHB drawbacks, it can be blended with other biodegradable polymers such as polylactic acid (PLA) and poly(butylene adipate-co-terephthalate) (PBAT), as well as plasticisers including acetyl tributyl citrate (ATBC) and tributyl citrate (TBC).

In fact, blending with other biopolymers has also proven effective. PHB has been combined with flexible polymers such as PLA, polycaprolactone (PCL), polybutylene adipate-co-terephthalate (PBAT), and thermoplastic starch (TPS) to improve flexibility and reduce brittleness. Gao [22] reported that PLA/PHB blends exhibited strong intermolecular interactions, with PHB reducing PLA’s glass transition temperature and increasing impact toughness by a factor of three. Chen [23] showed how PLA mechanical properties could be enhanced thanks to the plasticisation effect of innovative synthetised epoxy waste dimer acid methyl ester, leading to significantly enhanced mechanical properties, including an elongation at break of 72.6% and an impact strength of 8.2 kJ/m^2^, compared to neat PLA. Zytner [24] used reactive extrusion with triallyl isocyanurate (TAIC) and peroxide to compatibilise PHBV/PCL blends, achieving an elongation at break of 700%, which is much higher than neat PHBV. Phattarateera [25] showed that TPS improves tear strength in packaging films. Olonisakin [26] reviewed biodegradable blends and emphasised compatibilisation strategies to improve miscibility and mechanical performance.

Finally, another way to improve PHB weak points is the addition of plasticisers during the processing phase. Citrate plasticisers have been found to be very effective, as reported in many previous studies [27,28,29,30,31]. Particularly, the use of citrates such as acetyl tributyl citrate (ATBC) and tributyl citrate (TBC) has shown promise in enhancing flexibility and reducing brittleness, but these additives can migrate over time, potentially compromising long-term mechanical integrity and food safety [18,32].

Given these findings, it makes sense to study the specific combinations of PHB with PLA, PBAT, ATBC, and TBC.

## 2. Materials and Methods

### 2.1. Materials

The selected polymers in this work are poly(3-hydroxybutyrate) (PHB) Y3000P from Enmat, Ningbo City, China, poly(lactic acid) (PLA) Ingeo 2003D from NatureWorks, Minnetonka, MN, USA, poly(butylene-co-adipate)terephtalate (PBAT) PBE 006 extrusion grade from NaturePlast, Mondeville, France, all in pellet form.

According to the technical data sheet, the used PHB has an average molecular weight of 280,000 g × mol^−1^, a specific gravity of 1.25 g cm^−3^ and an MFI of 10–25 at 190 °C. PLA has an average molecular weight of 180,000 g mol^−1^, a specific gravity of 1.24 g × cm^−3^ and an MFI of 6 at 210 °C; the polymer mainly consists of L-isomer, with the D-isomer content lower than 4%. PBAT has an average molecular weight of 137,400 g × mol^−1^, a specific gravity of 1.26 g × cm^−3^ and an MFI of 4–6 at 190 °C.

The following plasticisers were also used: tributyl citrate CITROFOL BI (TBC) and acetyl-tributyl citrate CITROFOL BII (ATBC). TBC (chemical formula: C_18_H_32_O_7_) was purchased from BCD Chemie (Schellerdamm, Hamburg, Germany); it has a molecular weight of 360.4 g × mol^−1^. TBC is described as a clear, practically colourless, oily liquid. It is insoluble in water, freely soluble in alcohol, in isopropyl alcohol, in acetone and in toluene. Also, ATBC (chemical formula: C_20_H_34_O_8_) was purchased from BCD Chemie; it has a molecular weight of 402.48 g × mol^−1^ and has the same characteristics as TBC for colour and solubility in liquids.

### 2.2. Materials Preparation

The polymer pellets were subjected to overnight drying at 60 °C in an oven to eliminate any moisture content. The PHB and the additional biopolymer (PLA, PBAT) were mixed using a HAAKE Rheomix 600/610 from Thermo Scientific, Waltham, MA, USA, for 5 min at 180 °C. The rotational speed of the screw was set at a constant value of 40 revolutions per minute (rpm). Afterwards, plasticiser (TBC or ATBC) was added at a concentration of 20% by mass, and the mixing process was continued for another 10 min. The choice of adding the plasticisers at 20% by mass was supported by many previous studies [18,33,34]. Furthermore, it is the minimum amount required in order to lower the glass transition temperature of PLA under room temperature.

The compositions of the blends prepared are indicated in Table 1.

The properly mixed material was collected and then placed in a vented oven at 240 °C. When the temperature of the material reached 180 °C, the material was taken out of the oven and pressed in the hot press P7/91/PL, produced by the Italian company Campana S.r.l., Boretto, Italy, for 3 min, in order to obtain a thin film (dimensions of the film: 10 cm × 10 cm × 0.04 cm). The temperature of the plates of the hot press was set to 200 °C, and the pressure was set to 10 bar. Cooling was performed with air at ambient temperature.

No further conditioning of the films was carried out for subsequent testing.

Therefore, the maximum temperature experienced by the blends during processing is 180 °C. TGA analysis was done on the mixed and hot-pressed blends, showing that the onset temperature of degradation is 279 °C for mixed blends and 281 °C for hot-pressed blends, whereas the corresponding temperatures at a weight loss of 5% were 281 °C and 283 °C. Such small differences indicate that the effects of thermal degradation are case negligible under the processing conditions investigated.

### 2.3. Material Characterisation

#### 2.3.1. Thermal Analysis

Thermal analysis was performed using a Mettler Toledo 822 heat flow calorimeter manufactured by Mettler Toledo in Greifensee, Switzerland, under a dry nitrogen gas flow rate of 60 mL/min. Initially, samples were heated at 200 °C in order to eliminate the thermal history of the sample. Subsequently, cooling was performed from 200 °C to 0 °C with a cooling rate of 10 °C/min. Finally, the heating scan was carried out from 0 °C to 220 °C using a heating rate of 10 °C/min. Between each scan, an isothermal time of 2 min was set.

The cooling curve was used to determine the parameters for melt crystallisation, whereas the second heating curve was used to determine the usual parameters for cold crystallisation and melting.

The deconvolution of the melting signal for plasticised and non-plasticised blends was performed by nonlinear fitting of experimental data by a series of 2 to 4 Gaussian curves, depending on the observed number of melting peaks. In order to compare each of the deconvoluted melting enthalpies with melting enthalpies of neat polymers, the deconvoluted enthalpy is multiplied by a factor of two, accounting for the fact that each blend is composed of 50% PHB and 50% of the second polymer, either PLA or PBAT.

An example of the procedure for a PHB-PLA blend is as follows. Initially, the total melting peak (A_tot_) is deconvoluted by 4 Gaussian curves (A_i_), which provides the integral for each peak:

$$\frac{A_{tot}}{m_{PHB+PLA}}=\frac{A_{1}+A_{2}+A_{3}+A_{4}}{m_{PHB+PLA}}$$

However, in order to compare the area of any of the deconvoluted peaks with that of each polymer, we should consider normalisation by the mass of PHB or PLA:

$$\frac{A_{i}}{m_{PHB}}=\frac{A_{i}}{m_{PHB+PLA}}\times \frac{m_{PHB+PLA}}{m_{PHB}}=2\times \frac{A_{i}}{m_{PHB+PLA}}$$

$$\frac{A_{i}}{m_{PLA}}=\frac{A_{i}}{m_{PHB+PLA}}\times \frac{m_{PHB+PLA}}{m_{PLA}}=2\times \frac{A_{i}}{m_{PHB+PLA}}$$

Accounting for the fact that in any blend $m_{PHB}=m_{PLA}$.

Therefore, in the analysis of results, the sum of the areas reported from the deconvolution is only apparently twice the total area, resulting from the fact that the deconvoluted area is normalised by the weight of each polymer, whereas the total area is normalised by the total sample weight.

#### 2.3.2. Plasticisers Loss Test

Plasticiser loss tests were carried out using a vented Carbolite Oven, Hope, UK, keeping a temperature of 100 °C. Sample weights were measured at different times (0 h, 4 h, 8 h, 12 h, 24 h, 48 h, 72 h, 96 h, 120 h, 144 h, 168 h) in order to assess the plasticiser loss until constant weight was attained. Their weights were recorded using a precise weight balance (standard error of the weight balance ±0.01 g).

In order to evaluate the plasticiser loss percentage, the following equation was used:

$$Weight\;loss\;\left(\%\right)=\;\frac{m_{0}-m_{t}}{m_{0}}\times 100$$

where m_0_ is the initial sample mass (before the test), m_t_ is the sample mass at any time t.

#### 2.3.3. Mechanical Test

Tensile tests were performed on a LLoyd LR50K dynamometer, Bognor Regis, UK, according to ASTM D638 standard, using 5 mm × min^−1^ crosshead speed and 10 × 1 × 0.04 cm samples. For each measurement, 5 specimens were used to properly assess mechanical properties.

Young’s modulus (E) was calculated from the slope of the stress–strain curve in the linear part. Ultimate tensile strength (UTS) and its relative strain (ε_UTS_) were calculated in correspondence with the maximum stress.

Finally, strain at break (ε_b_) and strength at break (σ_b_) were calculated in correspondence with sample failure, while toughness was calculated from the integration of the stress–strain curves.

#### 2.3.4. Dynamic Mechanical Analysis

Dynamic mechanical analysis (DMA) tests were performed on PHB-PLA blends to assess their viscoelastic properties. An ARES rheometer from Rheometric Scientific was used in the torsion configuration. The samples were 40 mm long, 10 mm wide and 1 mm thick. The DMA tests were performed from 30 °C to 140 °C at a constant heating rate of 3 °C/min and a frequency of 1 Hz.

#### 2.3.5. Scanning Electron Microscopy

A scanning electron microscope (SEM) Zeiss EVO 40, Jena, Germany, equipped with a tungsten filament was used to evidence the fracture surfaces of previously traction-tested specimens. SEM pictures were taken at a magnification of ×250, which was considered sufficient for the investigation of the fractured surface.

## 3. Results and Discussion

### 3.1. Compatibility Analysis

#### 3.1.1. Interaction Radius from PHB

The compatibility of PHB with the different plasticisers was analysed using the interaction radius derived from Hansen Solubility Parameters (HSPs). The three components of the HSPs were determined following the Hoftyzer–Van Krevelen group contribution approach [35,36,37]. The Hansen Solubility Parameter of tested materials can be calculated using the following equation:

$$\delta =\sqrt{\delta_{d}^{2}{\;+\;\delta }_{p}^{2}\;+\delta_{H}^{2}}$$

where *δ*_*d*_ stands for the energy density resulting from dispersion bonds, *δ*_*p*_ is the energy derived from dipolar intermolecular forces, and *δ*_H_ stands for the energy received from hydrogen bonds between molecules.

Bagley et al. (1991) [38] reported that dispersion and polar interactions contribute similarly to overall cohesion, whereas hydrogen bonding exhibits a distinctly different behaviour. Accordingly, they proposed combining the dispersion and polar components into a single term called *δ*_v_, as can be shown in the following equation:

$$\delta_{v}=\sqrt{\delta_{d}^{2}\;{+\;\delta }_{p}^{2}}$$

This led to a diagram where *δ*_*v*_ can be displayed on the x-axis, and *δ*_H_ on the y-axis.

Finally, the interaction radius (IR) can be calculated using the following equation:

$$IR\;=\;\sqrt{{\left(\delta_{v}^{PHB}\;{-\;\delta }_{v}^{material}\right)}^{2}+{\left(\delta_{H}^{PHB}{-\delta }_{H}^{material}\right)}^{2}}$$

According to the Hansen Solubility Parameter theory, a smaller interaction radius suggests improved compatibility between the plasticiser and the polymer.

**Figure 1:**
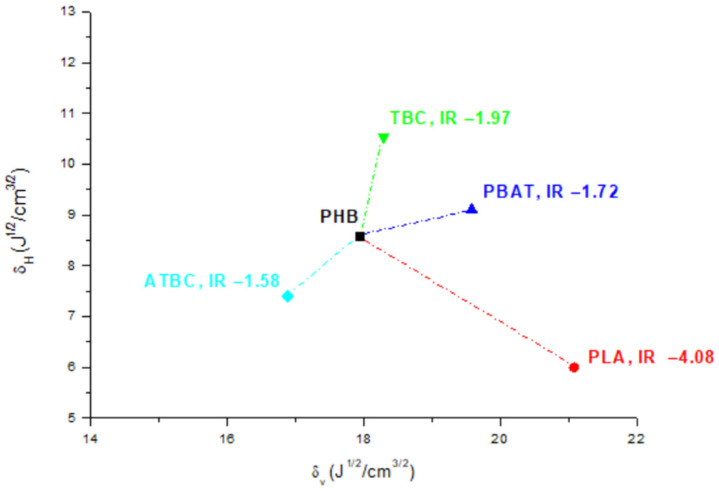
Interaction radius of the materials (PLA, PBAT, TBC, ATBC) with respect to PHB.

The Hansen Solubility Parameters (*δ*_d_, *δ*_*p*_, *δ*_H_, expressed in MPa^0.5^) used to evaluate the affinity between citrate plasticisers, biopolymers and PHB are represented in Table 2 [37,39,40]:

Figure 1 visually shows the calculated IR of the various materials with respect to PHB:

PHB exhibits a relatively strong affinity for citrate plasticisers, particularly ATBC, which shows the lowest interaction radius (1.58). It also demonstrates good compatibility with PBAT, as indicated by an interaction radius of 1.72. Conversely, PHB displays limited affinity with PLA, which is reflected in its comparatively large interaction radius.

#### 3.1.2. Interaction Radius from PHB/PLA and PHB/PBAT Blends

An alternative approach is to calculate the interaction radius of each plasticiser with respect to the polymer blend (i.e., PHB–PLA and PHB–PBAT). The Hansen Solubility Parameter of each polymer blend was calculated by simple additivity, assuming each blend contains 50% PHB and 50% PLA or PBAT; this reduces to averaging the HSPs of PHB with those of PLA or PBAT, respectively. Using these averaged values as reference points, the interaction radii for the citrate plasticisers can then be determined.

**Figure 2:**
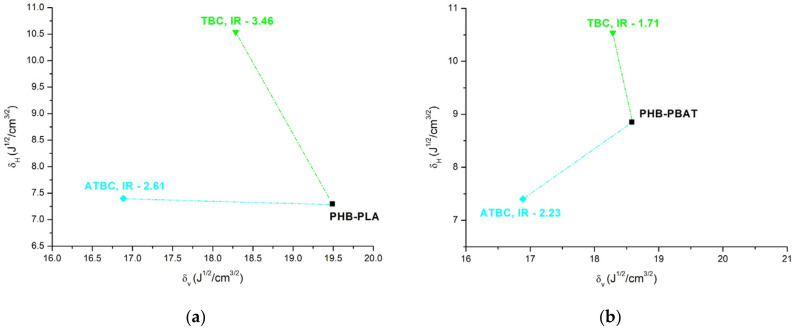
Interaction radius of (**a**) the PHB-PLA system with respect to TBC and to ATBC, and (**b**) the PHB-PBAT system with respect to TBC and ATBC.

Considering the HPSs presented in Table 2, the mean values are as follows (Table 3):

Figure 2a depicts the calculated IR between the PHB-PLA blend and citrate plasticisers, while Figure 2b shows the IR between the PHB-PBAT blend and the same plasticisers.

It is evident that the PHB–PBAT system exhibits stronger interactions with citrate plasticisers compared to the PHB–PLA system. In particular, the former shows good affinity for TBC, reflected by the lowest interaction radius of 1.71. Conversely, the PHB–PLA system appears to have comparatively better affinity for ATBC. However, its IR remains higher than that observed for the PHB–PBAT system.

### 3.2. Thermal Analysis

#### 3.2.1. Cooling

The thermal behaviour was first investigated for the neat polymer and the blends, and then for the plasticised polymers and blends, in order to systematically evaluate the effect of composition and plasticiser type on the crystallisation behaviour.

**Figure 3:**
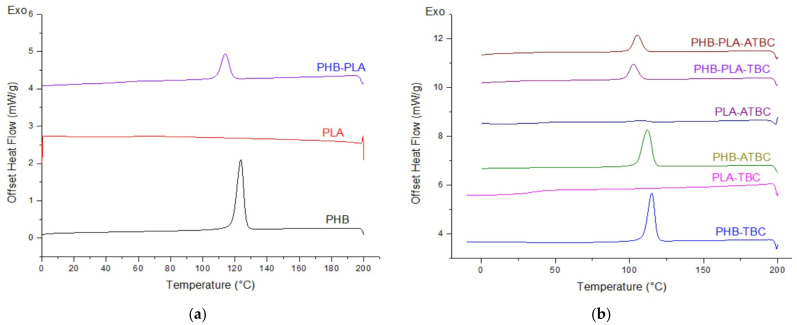
DSC cooling scans of (**a**) PHB, PLA and PHB-PLA blend, and (**b**) plasticised PHB, plasticised PLA and plasticised PHB-PLA blends.

Figure 3a shows the cooling thermograms of neat PHB, neat PLA and the PHB-PLA blend, while Figure 3b shows the thermograms for the same systems plasticised with TBC and ATBC.

**Figure 4:**
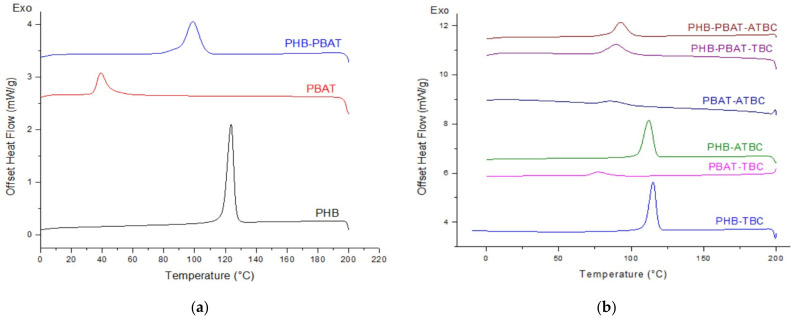
DSC cooling scans of (**a**) PHB, PBAT and PHB-PBAT blend, and (**b**) plasticised PHB, plasticised PBAT and plasticised PHB-PBAT blends.

Figure 4a shows the cooling thermograms of neat PHB, neat PBAT and PHB-PBAT blend, while Figure 4b shows the thermograms for the same systems plasticised with TBC and ATBC.

All DSC curves of the cooling cycle exhibited a distinct and single crystallisation exotherm for all samples where crystallisation takes place. In Figure 3a and Table 4, the crystallisation behaviour strongly depends on both the polymer matrix and the type of plasticiser. Neat PHB exhibits a high crystallisation temperature (T_c_ ≈ 123 °C) and high crystallisation enthalpy, confirming its strong tendency to crystallise rapidly upon cooling. In contrast, neat PLA does not crystallise under the adopted cooling conditions. The PHB-PLA blend shows a crystallisation enthalpy which is much lower than half of pure PHB, which involves a significant reduction in the crystallisation enthalpy attained by the latter.

In Figure 3b, it is shown that the addition of plasticisers significantly modifies the crystallisation behaviour of PHB: both TBC and ATBC increase the crystallisation enthalpy (ΔH_c_ up to 72.7 J/g) while shifting T_c_ to lower temperatures (115 °C for TBC and 112 °C for ATBC).

PLA plasticised by addition of TBC does not show any crystallisation peak, whereas a slight crystallisation is observed with ATBC (ΔH_c_ = 3.5 J/g, T_c_ = 108 °C), suggesting a marginal nucleating or mobility-enhancing effect of this plasticiser.

The addition of plasticisers to PHB-PLA restores crystallisation of PHB to some extent, as observed by comparison with the crystallisation enthalpies of the non-plasticised blend, although T_c_ is shifted to lower temperatures, confirming enhanced chain mobility but delayed crystal formation.

PBAT shows low-temperature crystallisation (T_c_ ≈ 39 °C); in the PHB–PBAT blend, the crystallisation enthalpy reported in Table 4 is 43 J/g, a value roughly equal to the theoretical crystallisation enthalpy obtained by the rule of mixtures with the measured enthalpies of neat PHB and PBAT, suggesting that high crystallinity of PHB and PBAT is retained.

The crystallisation of PBAT is significantly shifted to high temperatures upon plasticisation (T_c_ = 77 °C for TBC and about 86 °C for ATBC), also followed by a reduction in crystallisation enthalpy. This behaviour suggests that plasticisers promote earlier crystallisation in PBAT while limiting the extent of crystal growth.

In the PHB-PBAT blend, plasticisers reduce T_c_ from about 99 °C (observed for non-plasticised PHB-PBAT) to 90–93 °C. Also, it can be observed that plasticisers slightly reduce crystallisation enthalpy.

Overall, the results highlight that plasticisers primarily act by increasing chain mobility, with different effects depending on the polymer system: they delay PHB crystallisation while enhancing its extent; they induce limited crystallisation in PLA only in the presence of ATBC; and they shift PBAT crystallisation to higher temperatures while reducing its crystallinity.

Among the two plasticisers, ATBC generally shows a more pronounced effect in promoting crystallisation in blended systems, indicating better compatibility and more efficient modulation of phase behaviour.

Table 4 illustrates the numerical values obtained from the DSC cooling scans.

#### 3.2.2. Heating

During the second heating scan, the thermal transitions further highlight the different effects of TBC and ATBC on the individual polymers and on the blend systems.

**Figure 5:**
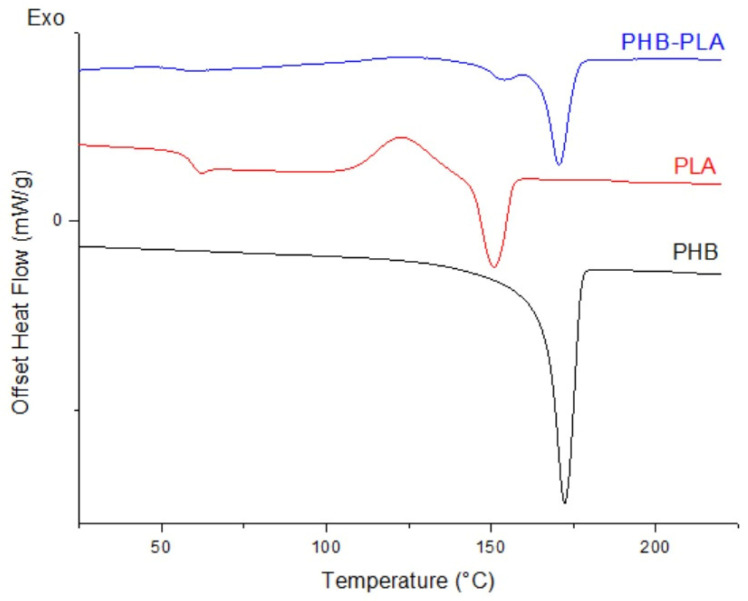
DSC heating scans of PHB, PLA and PHB-PLA blend.

Figure 5 displays the thermograms of neat PHB, neat PLA and the PHB-PLA blend.

Neat PHB does not exhibit cold crystallisation, confirming that crystallisation was already largely completed during cooling. One single melting peak is observed in the range 140–180 °C. Neat PLA, in contrast, shows an initial glass transition signal in the range 55–60 °C, followed by a marked cold crystallisation peak. In the absence of melt crystallisation, the subsequent melting peak is attributed to the cold-crystallised PLA. In fact, as reported in Table 5, melting enthalpy exactly corresponds to cold crystallisation enthalpy. PHB–PLA shows a cold crystallisation peak in the same range as the cold crystallisation peak of neat PLA. The cold crystallisation enthalpy of PHB-PLA is about half of the cold crystallisation enthalpy of neat PLA. However, since the former is calculated with respect to the total sample weight, normalisation with respect to PLA mass alone would provide the same enthalpy, indicating that the presence of PHB does not significantly interfere with PLA crystallisation.

In addition, the presence of two distinct melting peaks, the first corresponding to PLA melting and the second to PHB melting, clearly indicates the formation of a two-phase system. In fact, PHB crystallises during cooling, when PLA remains substantially amorphous; however, upon subsequent heating, PLA undergoes cold crystallisation, which occurs outside the crystalline domains of PHB, leading to the formation of a phase-separated structure.

An analysis of the enthalpies corroborates such a conclusion: the total melting enthalpy results from the sum of melt and cold crystallisation. By deconvolution, the melting enthalpies of the two peaks were calculated to be 23.2 J/g and 43.8 J/g, respectively. The first value roughly corresponds to the melting enthalpy of neat PLA, indicating that the degree of crystallinity attained by PLA during cold crystallisation is not influenced by the presence of PHB. The melting enthalpy of the second peak is about 43.8 J/g, which is a value much lower than that of neat PHB, and it confirms that the presence of PLA inhibits PHB melt crystallisation.

Results related to the cited thermograms are reported in Table 5 for the PHB and PLA blends.

**Figure 6:**
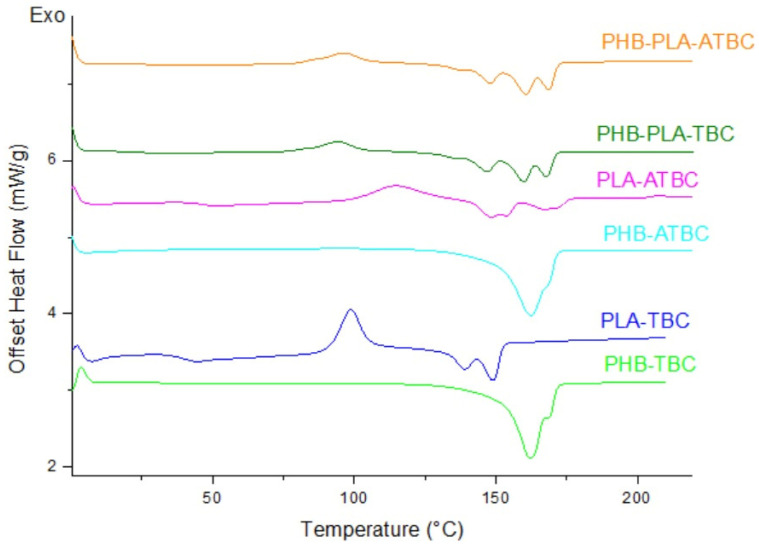
DSC heating scans of plasticised PHB, plasticised PLA and plasticised PHB-PLA blends.

The effect of the addition of plasticiser in PHB, PLA and PHB-PLA blends is reported in Figure 6.

For neat PHB, addition of TBC and ATBC results in a decrease in the melting temperature and an increase in melting enthalpy. As in the case of neat PHB, the melting enthalpy roughly corresponds to the melt crystallisation enthalpy, since no further crystals are formed during heating. Therefore, the increased melting enthalpy confirms the conclusion that a higher degree of crystallinity in plasticised PHB is attained during the cooling stage. The decrease in melting temperature observed for plasticised PHB, with T_m_ shifting from 172.4 °C for neat PHB to about 162 °C for both PHB–TBC and PHB–ATBC, is consistent with the decrease in equilibrium melting temperature brought by the addition of plasticiser, as provided by the Flory–Huggins theory [41,42].

In addition, plasticiser involves the presence of multiple peaks, which is due to recrystallisation phenomena occurring during heating. In fact, compared to neat PHB, the shift in the crystallisation peak for plasticised PHB indicates the formation of thinner crystals, which can undergo recrystallisation and lamellar thickening during heating. No clear effect of plasticiser type can be understood from the results of Figure 6.

For plasticised PLA, a downward shift in the glass transition with respect to PLA is initially observed. The addition of TBC involves a much lower T_g_ compared to ATBC, which is indicative of a better plasticising efficiency of the former. Following the glass transition, the plasticised PLA undergoes cold crystallisation. Even in this case, a significant difference between the two plasticisers can be understood: TBC involves a much faster cold crystallisation compared to ATBC, which again can be indicative of a better plasticising efficiency [43,44,45]. The following melting peak is a result of the structure evolution attained during cold crystallisation: thinner crystals formed at lower temperature for TBC-plasticised PLA melt at lower temperatures, compared to thicker crystals formed at higher temperatures for PLA-ATBC. Multiple melting peaks occur for both plasticised PLA systems as a consequence of recrystallisation effects.

In addition, for ATBC-plasticised PLA, a further melting peak in the range 160–175 °C indicates the existence of a second population of lamellar thicknesses. In agreement with results obtained from cooling curves, such lamellae correspond to the crystals formed during melt crystallisation.

For the plasticised PLA-PHB blends, a first glass transition is observed in a temperature range of 25.8 °C and 27.6 °C for TBC and ATBC, respectively. Such temperatures are much lower than those measured on plasticised PLA. Regarding the blend composition, it must be highlighted that 20phr of plasticiser is added relative to the total polymer content. However, plasticiser can be preferentially absorbed by any of the two polymers.

Therefore, starting from the Flory–Fox equation:

$$\frac{1}{T_{g\;plasticised\;PLA}}=\;\frac{w_{PLA}}{T_{g\;PLA}}+\frac{w_{plasticisers}}{T_{g\;plasticiser}}$$

The equation can be rearranged to give:

$$\frac{1}{T_{g\;plasticised\;PLA}}-\frac{1}{T_{g\;PLA}}=w_{plasticisers}\left(\frac{1}{T_{g\;plasticiser}}-\frac{1}{T_{g\;PLA}}\right)$$

In the absence of reliable data for T_g_ of TBC and ATBC, comparing the T_g_ of plasticised PLA (T_g1_) and plasticised PLA in PLA-PHB blends (T_g2_) leads to:

$$\frac{w_{plasticiser2}}{w_{plasticiser1}}=\frac{\frac{1}{T_{g2}}-\frac{1}{T_{g\;PLA}}}{\frac{1}{T_{g1}}-\frac{1}{T_{g\;PLA}}}=1.72$$

Therefore, the plasticiser absorbed by the PLA phase in PLA-PHB blends is higher than the plasticiser content in neat PLA. This indicates that during cooling, plasticiser is expelled from PHB crystalline domains, leading to an excess of plasticiser, which can be absorbed in the PLA phase.

After the glass transition, the plasticised blends show a cold crystallisation peak. Such a peak is shifted to lower temperatures compared to plasticised PLA and indicates that melt-crystallised PHB acts as a nucleating agent during the cold crystallisation of PLA.

For both plasticised blends, the melting profile shows four different peaks: interestingly, the first and the second peaks occur in correspondence with the melting peak of plasticised PLA, whereas the last two peaks occur within the melting range of plasticised PHB. This clearly indicates that, as in the case of non-plasticised PHB-PLA, a two-phase structure is formed, with PHB crystalline domains forming during the cooling stage and the PLA crystalline phase forming during the following heating stage. Even in this case, the deconvolution of the peaks was performed: for both plasticisers, the melting enthalpy resulting from the sum of the first and second peaks is estimated to be about 28 J/g, which roughly corresponds to the value of plasticised PLA. The sum of the enthalpies of the third and the fourth peaks is, in both cases, around 58–59 J/g, which is higher than the melting enthalpy of PHB in PHB-PLA blends, but much lower than the melting enthalpy of plasticised PHB.

Such results confirm the observation that PLA negatively affects PHB crystallisation: the addition of a plasticiser can, to some extent, enhance PHB crystallinity in the PHB-PLA systems, which, in any case, remains lower than that of plasticised PHB.

**Figure 7:**
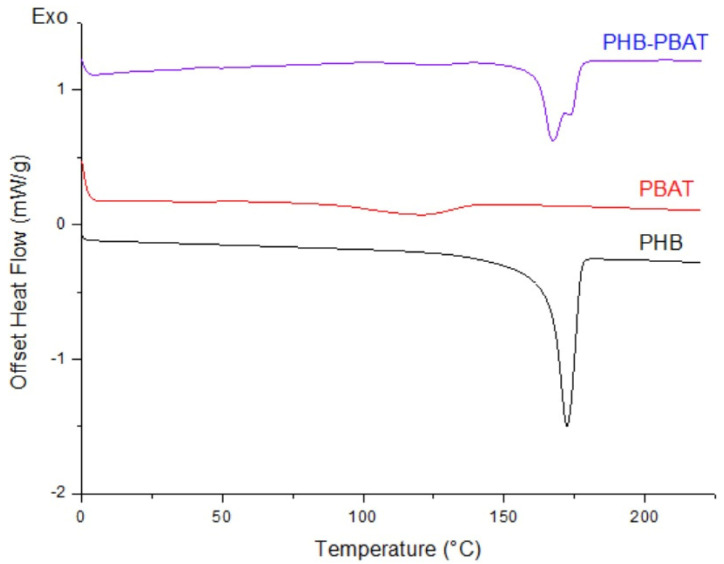
DSC heating scans of PHB, PBAT and PHB-PBAT blend.

Figure 7 displays the thermograms of neat PHB, neat PBAT and PHB-PBAT blend.

Results related to the cited thermograms are reported in Table 6 for the PHB and PBAT blends.

Neat PBAT shows a melting enthalpy of about 15 J/g, which roughly corresponds to its melt crystallisation enthalpy, at a temperature of about 120 °C. The blend PHB-PBAT shows a first melting peak within the melting range of PBAT and a second melting peak in the range of PHB melting. The melting profile of PHB in the PHB-PBAT blend shows the presence of two peaks, indicating recrystallisation effects, in agreement with cooling profiles, which showed the formation of thinner crystals at lower temperatures, when comparing PHA-PBAT with neat PHB. Deconvolution integrals are also reported in Table 6. The deconvolution area of the first peak is 4.4 J/g, much lower than that of PBAT, indicating that PBAT crystallisation is suppressed in the presence of PHB. In contrast, the sum of the deconvolution area of the second and third peaks is about 70 J/g, a value well in line with that of neat PHB, indicating that the presence of PBAT does not significantly affect the crystallisation behaviour of PHB.

**Figure 8:**
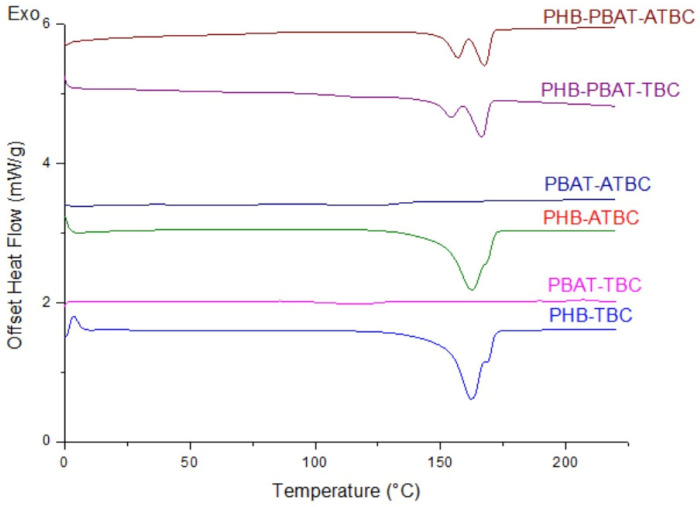
DSC heating scans of plasticised PHB, plasticised PBAT and plasticised PHB-PBAT blend.

The effect of the addition of plasticiser in PHB, PBAT and PHB-PBAT blends is reported in Figure 8.

For both plasticised PBAT samples, a melting peak is observed at around 120 °C, with a melting enthalpy of 5.9 J/g and 5.2 J/g for TBC and ATBC, respectively, which is much lower than that of neat PBAT, indicating that the presence of plasticiser reduces the crystallisation capacity of PBAT, in agreement with the results obtained from cooling curves.

For the plasticised PHB-PBAT blends, the melting peaks are present in the range of both PBAT and PHB melting. However, the area of the first deconvolution peak is much lower than that of neat PBAT, which confirms that the presence of PHB inhibits PBAT crystallisation. In contrast, the sum of second and third deconvoluted areas roughly corresponds to the melting enthalpy of neat PHB, indicating that addition of PBAT does not significantly modify the extent of PHB crystallisation.

### 3.3. Plasticisers Loss

**Figure 9:**
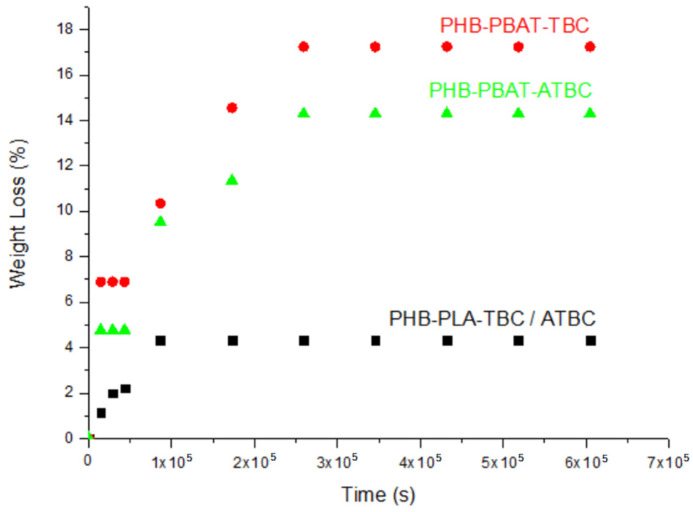
Weight loss associated with plasticiser migration for PHB-PLA-TBC, PHB-PLA-ATBC, PHB-PBAT-TBC and PHB-PBAT-ATBC.

Figure 9 shows the percentage weight loss of each blend. Please note that PHB-PLA-TBC and PHB-PLA-ATBC yielded the same results throughout the test, so their trend lines are superposed.

The weight-loss trend revealed distinct behaviours among the four polymeric systems. The PHB-PLA-based formulations (with both TBC and ATBC) exhibited very limited variations in mass, reaching a stable value already within the first hours of the test. This suggests that the amount of plasticiser able to migrate under the adopted conditions was negligible, and that the systems quickly attained equilibrium. Conversely, the PHB-PBAT blends showed a more evident reduction in weight, particularly in the presence of TBC, with a progressive loss observed up to 72 h before stabilisation. The corresponding ATBC-containing blend displayed an intermediate behaviour, with a slower and less pronounced decrease in mass. These results indicate that PBAT promotes a higher mobility of plasticisers compared to PLA, while the substitution of TBC with ATBC appears to mitigate the phenomenon, likely due to the lower volatility and higher molecular weight of ATBC.

The lower migration/volatility of ATBC compared to lower-molecular-weight citrate esters is discussed in the literature, which aligns with the data shown. ATBC is commonly reported as a low-volatility, heat-resistant citrate plasticiser, while migration and weight-loss tests at elevated temperature have shown measurable loss of citrate esters (including ATBC and other citrates) from PLA films [46]. This behaviour depends strongly on plasticiser molecular weight, polymer matrix and temperature [47]. Experimental and regulatory reports also documented occurrences of TBC in migration tests and identify migration as an application-relevant issue to consider when selecting citrate plasticisers.

The weight loss of non-plasticised samples under the same ageing conditions was negligible; therefore, data are not reported in Figure 9.

### 3.4. Mechanical Behaviour

**Figure 10:**
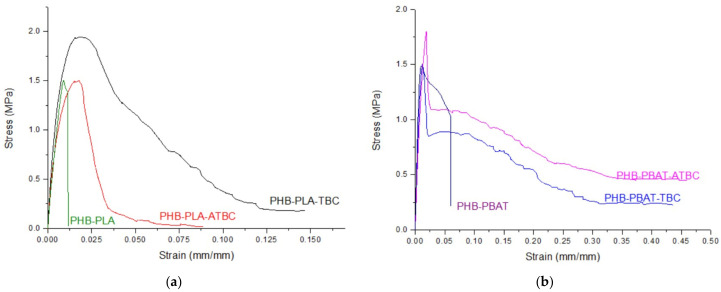
Stress–strain curves of the blends (**a**) PHB-PLA systems and (**b**) PHB-PBAT systems.

**Figure 11:**
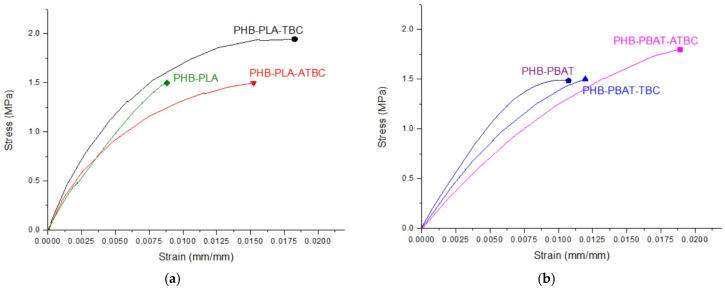
Stress–strain curves of the blends up to UTS (**a**) PHB-PLA systems and (**b**) PHB-PBAT systems.

In order to assess the mechanical behaviour of the prepared films, tensile tests were carried out as previously described. It is possible to appreciate the stress–strain curves in Figure 10, which show the entire stress–strain curve of the blends. Figure 11 shows an enlarged version of the curves, focusing on their UTS point.

Table 7 summarises the main mechanical results:

Mechanical properties obtained by tensile tests were also analysed by means of two-way analysis of variance (ANOVA). Two different sources of variation (factors) were considered:

- Factor 1: type of second biopolymer, PLA or PBAT, with two levels, and one degree of freedom;
- Factor 2: presence and type of plasticiser, with three levels (no plasticiser, TBC and ATBC), and two degrees of freedom.

The results for the ANOVA on tensile modulus are summarised in Table 8; the reported “*p*” values were compared with the confidence level, α = 0.05. The very low *p*-value obtained for Factor 1 indicates the relevant effect of the type of second biopolymer on the modulus of PHB blends.

Referring to the results of Table 7, it can be argued that the modulus of PHB-PBAT blends is much lower than that of PHB-PLA. Also, in Table 8, the low *p*-value obtained when considering Factor 2 indicates the relevance of the presence of plasticiser and plasticiser type on the modulus. Referring to the results of Table 7, it can be concluded that the presence of plasticiser significantly reduces the modulus of PHB blends;

Results reported in Table 9 and Table 10, relative to ANOVA of the strain at break and toughness, confirm the statistical relevance of both factors to these properties.

Finally, ANOVA was also performed on the tensile strength values; however, in this case, results (not reported) revealed that neither of the two factors had a statistically relevant effect on UTS.

Therefore, blends with PLA are characterised by statistically equivalent UTS values to blends with PBAT. Analogously, blends without plasticiser, with TBC and ATBC, are also characterised by statistically equivalent UTS values.

The mechanical characterisation of the investigated blends highlights clear differences associated with the type of plasticiser employed. In the PHB–PLA system, the use of TBC led to a slightly higher ultimate tensile strength (2.1 ± 0.8 MPa) and strain at break (16.1 ± 3.1%) compared to the ATBC counterpart. However, both PLA-based blends maintained relatively high stiffness, with Young’s modulus values above 300 MPa, suggesting that both the PHB and the PLA component largely preserved rigidity irrespective of the chosen plasticiser.

The non-plasticised blend (PHB-PLA) is characterised by brittle behaviour, showing a low value of strain at break (1.1 ± 0.1%) and, similarly, a very high value of Young’s modulus (4032.2 ± 112.4 MPa) compared to plasticised systems. In fact, thanks to the plasticisers’ effect, the tensile modulus dropped by 92% for the PHB-PLA-TBC and 90.6% for the PHB-PLA-ATBC blend. These results clearly show the plasticisation effect induced by the citrate plasticisers on the PHB-PLA systems.

In contrast, the PHB–PBAT blends showed a markedly different behaviour. While the UTS values were overall lower than those of the PHB–PLA samples, the incorporation of PBAT resulted in a dramatic improvement in ductility. The PHB–PBAT–ATBC blend, in particular, reached a strain at break of 67.8 ± 21.2%, far exceeding all other formulations. This enhanced deformability was accompanied by a significant reduction in stiffness, with the Young’s modulus dropping to values around 150 MPa, highlighting the strong toughening effect of PBAT and the high efficiency of ATBC in further promoting chain mobility. The PHB–PBAT–TBC blend also exhibited improved elongation at break compared to PLA-based systems, but to a lower extent (41.1 ± 4.5%) compared to the same blend plasticised with ATBC (67.8 ± 21.2), confirming a more moderate plasticising action of TBC than ATBC on PHB-PBAT systems.

Also in that case, the non-plasticised blend showed lower properties compared to plasticised ones, with lower values of elongation at break (6.0 ± 2.2%) and higher stiffness, as evidenced by the higher Young’s modulus (1366.2 ± 41.9 MPa). In fact, thanks to the plasticisation effect induced by TBC and ATBC, the elongation at break increased by 68.1% for the former and 35.1% for the latter, while the modulus dropped by 89.4% and 88.2%, respectively.

From a comparative perspective, these findings suggest that ATBC is a more effective plasticiser than TBC in the PBAT-containing blends, where the synergistic effect between the inherently ductile PBAT phase and ATBC maximises extensibility and drops the modulus. These findings are consistent with the IR and HSP previously discussed, where the distance between PHB and ATBC was only 1.58, showing a good affinity between them, while the distance between PHB and TBC was 1.97.

Conversely, when combined with PLA, ATBC appears to have a more moderate toughening effect compared to TBC, as highlighted by the lower elongation at break and the slightly higher modulus.

Finally, the toughness of the blends was calculated by integration of the stress–strain curves. The results, expressed in kJ/m^3^, show that the PHB-PBAT blends exhibit significantly higher toughness compared to the corresponding PHB-PLA systems. Specifically, the use of ATBC as a plasticiser in the PHB-PBAT-ATBC blend further enhances the toughness compared to TBC in the PHB-PBAT-TBC blend, reaching the highest value of 338.7 kJ/m^3^.

Also in that case, the non-plasticised blends show lower toughness values compared to plasticised ones: this is a direct result of the effectiveness of TBC and ATBC on the improved ductility of the systems.

### 3.5. Dynamic Mechanical Analysis

**Figure 12:**
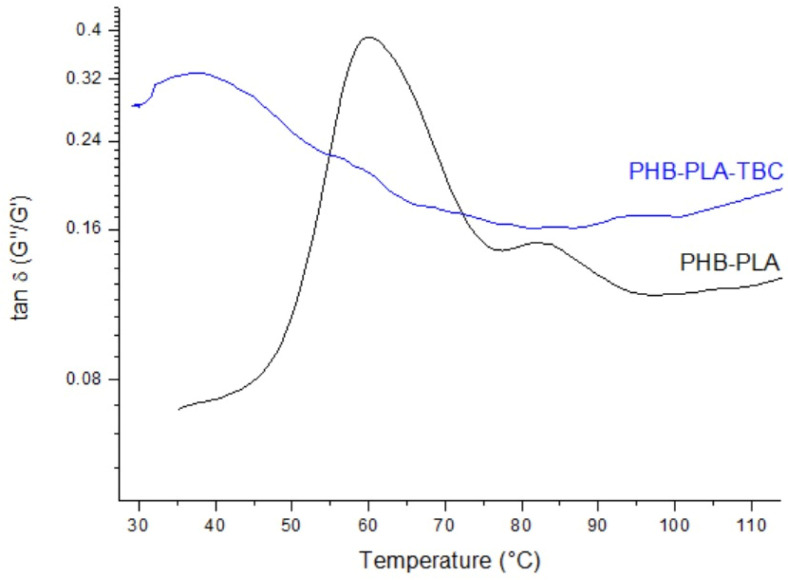
DMA curves of PHB-PLA and PHB-PLA-TBC.

The viscoelastic response of the investigated systems was further assessed by analysing the tan δ curves. As can be seen from Figure 12, the position of the tan δ peak clearly confirms the effect of plasticisation on the PLA phase within the blends. The non-plasticised PHB-PLA sample shows the main relaxation peak in the range of 50–60 °C, while the PHB-PLA-TBC and PHB-PLA-ATBC blends exhibit a pronounced shift in the peak toward lower temperatures, around 20–25 °C. This displacement indicates a substantial reduction in the PLA glass transition temperature, consistent with increased chain mobility induced by the plasticiser.

These findings are in good agreement with the DSC results, which showed the same trend in the glass transition temperature upon plasticiser addition, confirming the effectiveness of TBC and ATBC in promoting chain mobility.

Finally, the tan δ curve of PHB-PLA-ATBC blend is not reported in Figure 12 because it is essentially superimposable on that of PHB-PLA-TBC.

### 3.6. Scanning Electron Microscopy

SEM images were acquired on the fracture surfaces of tensile-tested specimens in order to further investigate the mechanical response of the different blends. At a magnification of 250×, the micrographs clearly highlight the distinction between brittle and ductile fracture behaviours.

**Figure 13:**
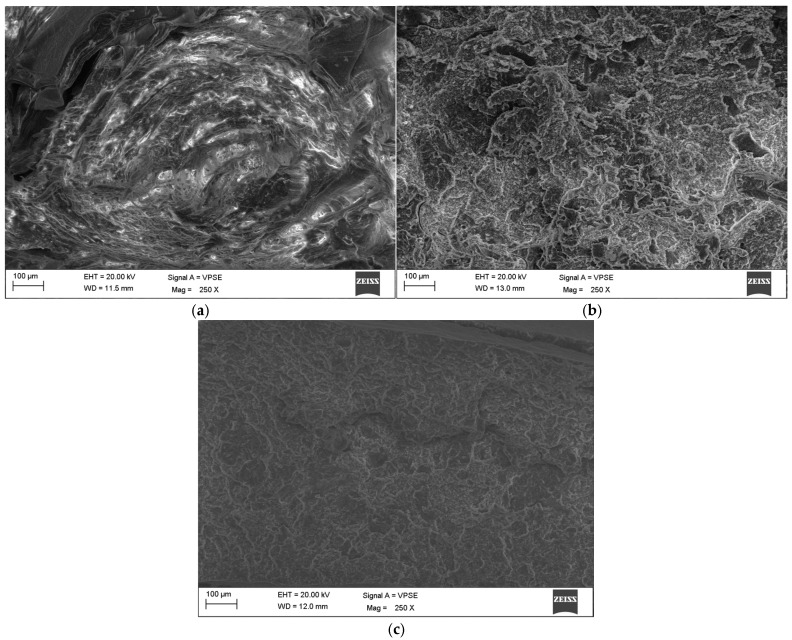
SEM images of (**a**) PHB-PLA, (**b**) PHB-PLA-TBC and (**c**) PHB-PLA-ATBC.

In particular, from the SEM pictures of the PHB-PLA-based systems presented in Figure 13, consistent with the mechanical properties previously discussed, it is possible to observe a brittle failure mode, as evidenced by the relatively smooth and planar fracture surfaces. Such morphology is typically associated with limited plastic deformation and rapid crack propagation, both of which are characteristic of brittle materials.

**Figure 14:**
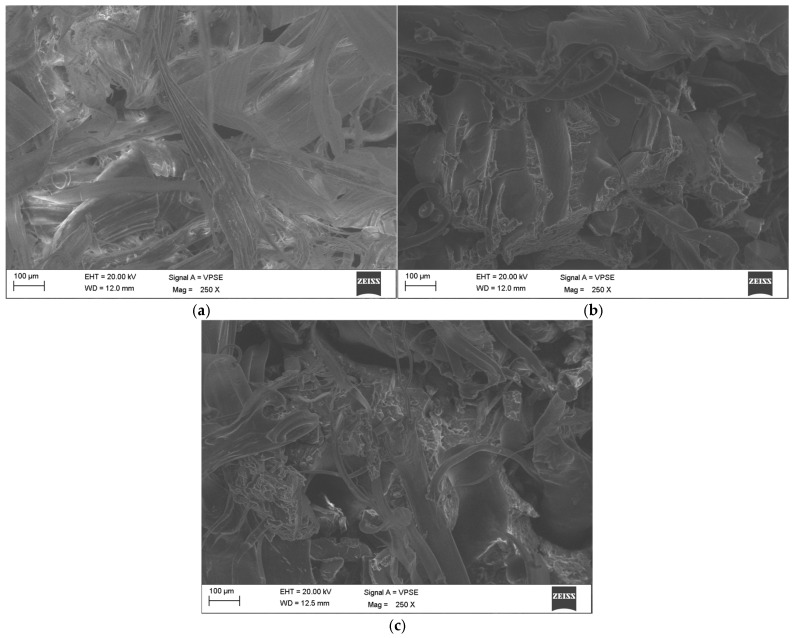
SEM images of (**a**) PHB-PBAT, (**b**) PHB-PBAT-TBC and (**c**) PHB-PBAT-ATBC.

Conversely, the PHB-PBAT systems, whose fracture surfaces are shown in Figure 14, show a markedly ductile fracture behaviour, with rough and irregular surfaces decorated by numerous filaments and fibrillar structures, which are indicative of extensive plastic deformation and energy dissipation during fracture.

Moreover, in the PHB-PBAT blends, it is possible to observe phase separation between PHB and PBAT, as demonstrated by the coexistence of areas displaying a brittle-like morphology, with planar surfaces, and regions showing a ductile response, characterised by the presence of PBAT-related filaments. This observation is also consistent with the DSC results, which had already suggested phase separation in the PHB-PBAT systems.

### 3.7. Thermogravimetric Analysis

Thermogravimetric measurements were performed on the PHB-PLA-TBC blend immediately after mixing and again on the films subsequently produced by hot pressing. This approach was adopted because the hot-pressed films had been subjected to two thermal cycles: the first during material preparation by mixing, and the second during film fabrication by hot pressing. Therefore, TGA analysis was carried out to verify whether thermal degradation occurred in PHB and PLA, which are the biopolymers most sensitive to thermal decomposition.

The samples were analysed over a temperature range from 25 °C to 900 °C, a condition selected to ensure complete thermal degradation of the material. As shown in the figure, the maximum weight loss was reached at about 370–380 °C, corresponding to a mass loss of approximately 25%. Importantly, within the processing temperature window used both during mixing and hot pressing (180 °C), no significant weight loss was detected. The first appreciable degradation event occurred only above 300 °C. These results therefore indicate that the processing conditions employed did not induce any relevant thermal degradation in the investigated samples.

## 4. Conclusions

The present work systematically investigated the effect of the addition of a citrate plasticiser on the thermal, morphological, and mechanical properties of PHB–PLA and PHB–PBAT blends. Overall, both TBC and ATBC significantly influenced the behaviour of the investigated systems, modifying crystallisation, melting, glass transition, and fracture characteristics depending on the polymeric matrix.

DSC analysis confirmed the phase-separated nature of both blend systems. In PHB–PLA blends, a two-phase structure was observed, with PHB crystallising upon cooling while PLA remained predominantly amorphous. The addition of plasticiser altered the characteristic crystallisation behaviour, generally shifting the relevant thermal transitions to lower temperatures. The results also suggest that during PHB melt crystallisation, the plasticiser is primarily absorbed by the amorphous PLA phase, which likely contributes to improved ductility and toughness. A similar phase-separated morphology was observed for PHB–PBAT blends, as shown by the well-defined crystallisation and melting peaks of the two components. In both systems, the presence of the plasticisers reduced the characteristic temperatures as well as the crystallisation and melting enthalpies compared with the corresponding unplasticised blends.

DMA results were fully consistent with the DSC findings, confirming the plasticiser-induced decrease in the glass transition temperature of the PLA phase in PHB–PLA systems. This reduction was reflected in the mechanical response of the blends, leading to a substantial decrease in stiffness and a marked increase in ductility. In particular, TBC was more effective in improving the ductility and toughness of PHB–PLA blends, whereas ATBC provided superior performance in PHB–PBAT systems.

The mechanical testing clearly demonstrated the remarkable ability of both plasticisers to make the blends significantly more ductile than their unplasticised counterparts. This effect was especially evident in the pronounced reduction in Young’s modulus and the increase in elongation at break. SEM observations supported these findings by showing brittle fracture surfaces in PHB–PLA blends and ductile fracture features in PHB–PBAT systems, together with clear evidence of phase separation.

Finally, TGA results showed that no appreciable thermal degradation occurred within the processing temperature window used for mixing and hot pressing, confirming the thermal stability of the materials under the adopted fabrication conditions. The accelerated loss tests further demonstrated that plasticiser volatility strongly depends on both the polymeric matrix and the citrate ester used. PHB–PLA systems showed negligible mass loss, whereas PBAT-based blends exhibited a more pronounced release, particularly for TBC. Replacing TBC with ATBC reduced the extent of migration, in agreement with the lower volatility and greater stability of ATBC reported in the literature.

Overall, these findings highlight the strong interplay between thermodynamic compatibility, thermal transitions, migration behaviour, fracture morphology, and mechanical performance, providing useful guidelines for the design of citrate-plasticised PHB-based biodegradable materials.

## Figures and Tables

**Table 1:** Compositions of blends.

	PHB (%)	PLA (%)	PBAT (%)	TBC (phr)	ATBC (phr)
**PHB-PLA-TBC**	50	50	0	20	0
**PHB-PLA-ATBC**	50	50	0	0	20
**PHB-PBAT-TBC**	50	0	50	20	0
**PHB-PBAT-ATBC**	50	0	50	0	20

**Table 2:** Hansen Solubility Parameters for PHB, PLA, PBAT, TBC and ATBC.

	*δ*_*p*_	*δ*_d_	*δ*_H_
**PHB**	9.0	15.5	8.6
**PLA**	9.9	18.6	6.0
**PBAT**	4.83	18.97	9.1
**TBC**	5.03	17.58	10.54
**ATBC**	2.5	16.7	7.4

**Table 3:** Hansen Solubility Parameters for the systems PHB-PLA, PHB-PBAT, TBC, and ATBC.

	*δ*_*p*_	*δ*_d_	*δ*_H_
**PHB-PLA**	9.45	17.05	7.3
**PHB-PBAT**	6.92	17.24	8.85
**TBC**	5.03	17.58	10.54
**ATBC**	2.5	16.7	7.4

**Table 4:** DSC parameters derived from cooling scan.

	ΔH_c_ (J/g)	T_c_ (°C)
**PHB**	59.9	123.7
**PLA**	-	-
**PHB-PLA**	22.6	114.0
**PHB-TBC**	72.8	115.1
**PHB-ATBC**	69.7	112.2
**PLA-TBC**	-	-
**PLA-ATBC**	3.5	108.2
**PHB-PLA-TBC**	29.4	102.8
**PHB-PLA-ATBC**	29.0	105.5
**PBAT**	21.0	39.1
**PHB-PBAT**	43.5	98.9
**PBAT-TBC**	10.9	77.0
**PBAT-ATBC**	11.8	85.9
**PHB-PBAT-TBC**	35.2	89.6
**PHB-PBAT-ATBC**	37.6	92.8

**Table 5:** DSC parameters derived from the second heating scan for PHB and PLA.

				Deconvolution Results								
	ΔH_cc_ (J/g)	T_cc_ (°C)	ΔH_m_ (J/g)	T_m1_ (°C)	A_1_ (J/g)	T_m2_ (°C)	A_2_ (J/g)	T_m3_ (°C)	A_3_ (J/g)	T_m3_ (°C)	A_4_ (J/g)	T_g_ (°C)
		*2nd Heating*										
**PHB**	-	-	−70.4	172.4	-	-	-	-	-	-	-	-
**PLA**	22.3	122.9	−22.7	150.8	-	-	-	-	-	-	-	58.9
**PHB-PLA**	10.6	125.3	−34.2	153.9	−23.2	170.6	−43.8	-	-	-	-	55.8
**PHB-TBC**	-	-	−81.3	162.3	-	-	-	-	-	-	-	-
**PHB-ATBC**	-	-	−77.6	162.4	-	-	-	-	-	-	-	-
**PLA-TBC**	29.2	98.9	−29.7	139.3	−40.6	149.1	−20.1	-	-	-	-	38.7
**PLA-ATBC**	24.4	114.9	−26.9	148.7	−37.8	154.3	−14.2	167.5	-	-	-	45.8
**PHB-PLA-TBC**	14.3	94.8	−44.1	136.8	−5.2	147.1	−23.4	160.0	−43.8	167.9	−14.2	25.8
**PHB-PLA-ATBC**	14.1	96.3	−47.7	138.2	−4.2	148.1	−25	160.7	−40.2	168.6	−17.8	27.6

**Table 6:** DSC parameters derived from the second heating scan for PHB and PBAT.

	ΔH_m_ (J/g)	T_m1_ (°C)	Deconv. (J/g)	T_m2_ (°C)	Deconv. (J/g)	T_m3_ (°C)	Deconv. (J/g)
**PHB**	−70.4	172.4	-	-	-	-	-
**PHB-TBC**	−81.3	162.3	-	-	-	-	-
**PHB-ATBC**	−77.6	162.4	-	-	-	-	-
**PBAT**	−15.1	120.9	-	-	-	-	-
**PBAT-TBC**	−5.9	121.1	-	-	-	-	-
**PBAT-ATBC**	−5.2	122.3	-	-	-	-	-
**PHB-PBAT**	−39.7	125.6	−4.4	167.6	−43.4	173.5	−27.4
**PHB-PBAT-TBC**	−33.1	120.4	−3.0	154.3	−32.8	166.2	−30.8
**PHB-PBAT-ATBC**	−37.4	118.8	−4.0	156.9	−34.8	167.1	−35.8

**Table 7:** Mechanical parameters derived from uniaxial tensile tests for the proposed blends.

	UTS (MPa)	ε_UTS_ (%)	ε_b_ (%)	E (MPa)	Toughness (kJ/m^3^)
**PHB-PLA**	1.5 ± 0.2	1.0 ± 0.2	1.1 ± 0.1	4032.2 ± 112.4	11.6
**PHB-PBAT**	1.5 ± 0.4	1.1 ± 0.1	6.0 ± 2.2	1366.2 ± 41.9	73.0
**PHB-PLA-TBC**	2.1 ± 0.8	1.5 ± 0.6	16.1 ± 3.1	320.4 ± 99.2	126.1
**PHB-PLA-ATBC**	1.8 ± 0.4	1.3 ± 0.6	8.2 ± 1.8	380.0 ± 85.3	46.8
**PHB-PBAT-TBC**	1.3 ± 0.1	1.5 ± 0.9	41.1 ± 4.5	161.8 ± 18.6	221.5
**PHB-PBAT-ATBC**	1.8 ± 0.1	2.5 ± 0.7	67.8 ± 21.2	145.5 ± 7.9	338.7

**Table 8:** Analysis of variance for the tensile moduli of the non-plasticised and plasticised blends.

Factor	DOF	SS	MS	F	*p*
**Factor 1: second biopolymer**	1	7.80 × 10^6^	7.80 × 10^6^	1.47 × 10^3^	4.8 × 10^−23^
**Factor 2: Plasticiser and plasticiser type**	2	3.99 × 10^7^	2.00 × 10^7^	3.76 × 10^3^	1.06 × 10^−30^
**Error**	24	1.27 × 10^5^	5.3 × 10^3^	-	-
**Total**	29	5.80 × 10^7^	-	-	-

**Table 9:** Analysis of variance for the stress at break of the non-plasticised and plasticised blends.

Factor	DOF	SS	MS	F	*p*
**Factor 1: second biopolymer**	1	6.68 × 10^3^	6.68 × 10^3^	8.22 × 10^1^	3.22 × 10^−9^
**Factor 2: Plasticiser and plasticiser type**	2	6.34 × 10^3^	3.17 × 10^3^	3.90 × 10^1^	2.85 × 10^−8^
**Error**	24	1.95 × 10^3^	8.12 × 10^1^	-	-
**Total**	29	1.88 × 10^4^	-	-	-

**Table 10:** Analysis of variance for the toughness of the non-plasticised and plasticised blends.

Factor	DOF	SS	MS	F	*p*
**Factor 1: second biopolymer**	1	1.68 × 10^5^	1.68 × 10^5^	1.08 × 10^3^	1.79 × 10^−21^
**Factor 2: Plasticiser and plasticiser type**	2	1.34 × 10^5^	6.71 × 10^4^	4.33 × 102	1.49 × 10^−19^
**Error**	24	3.72 × 10^3^	1.55 × 10^2^	-	-
**Total**	29	3.83 × 10^5^	-	-	-

## Data Availability

The original contributions described as a result of this study are included in the article. Further inquiries can be directed to the corresponding author.
